# Automatic yield-line analysis of slabs using discontinuity layout optimization

**DOI:** 10.1098/rspa.2014.0071

**Published:** 2014-08-08

**Authors:** Matthew Gilbert, Linwei He, Colin C. Smith, Canh V. Le

**Affiliations:** 1 Department of Civil and Structural Engineering, University of Sheffield, Sir Frederick Mappin Building, Mappin St., Sheffield S1 3JD, UK; 2 Department of Civil Engineering, International University - VNU HCMC, Ho Chi Minh City, Vietnam

**Keywords:** slabs, plates, plasticity, limit analysis, layout optimization, yield-line analysis

## Abstract

The yield-line method of analysis is a long established and extremely effective means of estimating the maximum load sustainable by a slab or plate. However, although numerous attempts to automate the process of directly identifying the critical pattern of yield-lines have been made over the past few decades, to date none has proved capable of reliably analysing slabs of arbitrary geometry. Here, it is demonstrated that the discontinuity layout optimization (DLO) procedure can successfully be applied to such problems. The procedure involves discretization of the problem using nodes inter-connected by potential yield-line discontinuities, with the critical layout of these then identified using linear programming. The procedure is applied to various benchmark problems, demonstrating that highly accurate solutions can be obtained, and showing that DLO provides a truly systematic means of directly and reliably automatically identifying yield-line patterns. Finally, since the critical yield-line patterns for many problems are found to be quite complex in form, a means of automatically simplifying these is presented.

## 1. Introduction

The yield-line method is a long established and highly effective means of estimating the ultimate load-carrying capacity of slabs and plates. The term ‘yield-line’ was coined by Ingerslev [1], with a comprehensive theory developed by Johansen [2], and, in parallel, by Gvozdev [3]. The upper bound status of the method within the context of the then emerging plastic theories of structural analysis was later confirmed by others (e.g. [4,5]). The method traditionally involves postulating a collapse mechanism which is compatible with the boundary conditions and then using the principle of virtual work to compute the ultimate load, or ‘load factor’.

For certain special cases, it has been possible to calculate provably exact failure load factors (e.g. Fox [6] established the exact solution for the case of a uniformly loaded fixed square slab). However, in the case of most real-world geometrical configurations exact load factors are not available. In such cases, unless the critical yield-line pattern has been identified, the computed load factor will over-estimate the true load factor. While lower bound methods can be used to bound the load factor from below, the gap between a yield-line solution and a solution obtained using common hand-based lower bound analysis methods (e.g. the strip method proposed by Hillerborg [7], which simplifies the problem by allowing analyst/designer to select load paths while ignoring twisting moments) will typically be found to be quite wide. This situation is clearly unsatisfactory and has undoubtedly limited the extent to which hand-based yield-line analysis is used in practice.

Consequently, various computational methods have been applied to the problem over the past few decades. For example, Anderheggen & Knopfel [8] were among the first to apply finite-element limit-analysis techniques to slabs, showing that rigorous lower bound solutions could be obtained providing a suitable element formulation was employed. More recently, it has been demonstrated that nonlinear optimization [9] and the second-order cone programming techniques [10–12] can be applied, obviating the need to linearize the yield surface. Meshless (element-free Galerkin) methods have also been applied to slab problems, and reasonably good approximations of the collapse load factor can be obtained rapidly [13]. However, despite the promise of such methods, they have not found their way into routine engineering practice and at present practising engineers typically have to instead rely on potentially cumbersome iterative elasto-plastic analysis methods. Furthermore, since finite-element (and meshless) methods are concerned with treatment of an underlying continuum mechanics problem, these methods do not directly identify patterns of yield-lines, though in many cases these can subsequently be inferred from the output.

To address this, computational methods capable of explicitly identifying yield-lines have also been developed in parallel. For example, Chan [14], and later workers such as Munro & Da Fonseca [15] and Balasubramanyam & Kalyanaraman [16], proposed (very similar) methods in which potential yield-lines are placed at the boundaries of rigid elements arranged in a finite-element mesh. This permits linear programming (LP) to then be used to identify the most critical layout of yield-lines. While available computing resources of the time meant that only relatively coarse meshes could be treated, the most significant problem is sensitivity of the results obtained to the chosen initial mesh layout, with the consequence that refining the mesh alone does not necessarily lead to an improved estimate of the collapse load factor. This, for example, means that when using a structured triangular mesh, however fine, it is impossible to accurately simulate a fan-type mechanism. Numerous attempts to overcome this fundamental problem have been made, for example, by subsequently changing the topology of the initial rigid finite-element mesh through the use of geometry optimization or other techniques (e.g. [17–19]), but no fully satisfactory solution to the problem has been found. (This was also the conclusion of Johnson [20], who, after many years work in the field, asserted that the upper bound problem was simply ‘too difficult’ to solve computationally.) A possible way round this was recently put forward by Jackson [21] and Jackson & Middleton [22], who proposed that the lower bound solution could be used to suggest the form of the yield-line solution. Promising results were presented, but the procedure involves both a manual interpretation step and a potentially problematic and time-consuming nonlinear optimization step, suggesting that a truly systematic means of identifying yield line patterns had yet to be found.

However, the popularity of application-specific yield-line analysis tools, for example the *COBRAS* reinforced concrete bridge assessment tool developed at the University of Cambridge, and which involves automatically searching through a library of possible yield-line failure mechanisms [23], indicates that a systematic yield-line method would undoubtedly find widespread application. Furthermore, a 2004 industry report reiterated the potential economic benefits of using yield-line design, despite the fact that at present the analysis must by necessity be performed by hand [24]. In the report, it is recommended that, because a hand analysis may not lead to identification of the most critical mechanism, a 10% margin of error (safety factor) should pragmatically be assumed. However, the basis for this particular value is not entirely clear, and the fact that a factor of this sort is needed at all is clearly not entirely satisfactory.

In this paper, the upper bound problem will be revisited using a ‘discontinuous’ rather than continuum analysis approach, on the surface similar to the methods proposed by Chan [14], Munro & Da Fonseca [15] and others. However, the significant difference here is that by formulating the problem in terms of *discontinuities* rather than *elements,* a very much wider range of failure modes will be able to be identified, thereby overcoming the sensitivity to the initial mesh layout encountered when using previously proposed methods. Furthermore, rather than initially considering the yield-line analysis problem directly, as most others have done (with only limited success), the procedure described in this paper was developed following a conjecture that there existed a direct analogy between the layout of bars in optimum trusses and the layout of yield-lines in slabs, since such an analogy had been identified in the case of in-plane plasticity problems [25]. As the problem formulation is somewhat different in this case, the original sequence of development is also preserved in this paper, with the nature of the analogy examined initially.

## 2. Analogy between optimal layouts of truss bars and yield-lines

### (a) Background

The analogy between the compatibility requirements of yield-line patterns and the equilibrium requirements of trusses appears to have been identified comparatively recently [26]. This finding is of interest since numerical layout optimization techniques have been applied to the problem of identifying optimal trusses for several decades (e.g. [27,28]). Furthermore, the efficiency of such methods have dramatically increased recently, with the advent of modern interior point LP solvers and also the application of adaptive refinement procedures [29]. Thus, layout optimization problems containing several billion potential connections between nodes (i.e. bars or yield-lines in this case) can now be solved on current generation personal computers.

However, while Denton [26] showed that a truss corresponding to a compatible yield-line pattern must have at least one state of self-stress (or ‘degree of redundancy’), it can be shown that there must always exist a statically determinate optimum solution for the single load case truss layout optimization problem. This makes the analogy perhaps less immediately obvious than that identified between discretized optimal truss layouts and the critical arrangement of slip-lines in plane plasticity problems [25]; in the latter case, many important plane plasticity problems have patterns of slip-lines defining the failure mechanism which correspond to the layouts of bars in statically determinate trusses. Furthermore, it is not immediately obvious how issues such as the presence of distributed out-of-plane live loading can be treated using the type of procedure used to identify optimal truss layouts (such loading is obviously often present in slab problems, but is absent from the basic truss layout optimization problem). To investigate this further, various approximate-discretized LP truss layout optimization formulations will now be considered.

### (b) Layout optimization of trusses: linear programming formulations

First, consider a potential planar design domain which is discretized using *n* nodes and *m* potential nodal connections (truss bars). The classical ‘equilibrium’ plastic truss layout optimization formulation for a single load case is defined in equation (2.1) as follows (after [27]):

2.1 $$\begin{array}{rl} \text{min }\; & V={\text{c}}^{T}\text{q}\\ \text{subject to:}\; & \text{Bq}=\text{f}\\ & \text{q}\geq \text{0}, \end{array}$$

where *V* is the total volume of the structure, $q^{T}=\{q_{1}^{+},q_{1}^{-},q_{2}^{+},q_{2}^{-}\ldots q_{m}^{-}\}$, and $q_{i}^{+},q_{i}^{-}$ are the tensile and compressive forces in bar *i*; **c**^*T*^={*l*_1_/*σ*^+^_1_,*l*_1_/*σ*^−^_1_,*l*_2_/*σ*^+^_2_,*l*_2_/*σ*^−^_2_…*l*_*m*_/*σ*^−^_*m*_}, where *l*_*i*_, *σ*^+^_*i*_ and *σ*^−^_*i*_ are, respectively, the length and tensile and compressive yield stress of bar *i*. **B** is a suitable (2*n*×2*m*) equilibrium matrix containing direction cosines and $f^{T}=\{f_{1}^{x},f_{1}^{y},f_{2}^{x},f_{2}^{y}\ldots f_{n}^{y}\}$ where $f_{j}^{x}$ and $f_{j}^{y}$ are the *x* and *y* components of the external load applied to node *j* ( *j*=1…*n*). The presence of supports at nodes can be accounted for by omitting the relevant terms from **f**, together with the corresponding rows from **B**. This problem is in a form which can be solved using linear optimization, with the bar forces in **q** being the LP variables.

**Figure 1.**
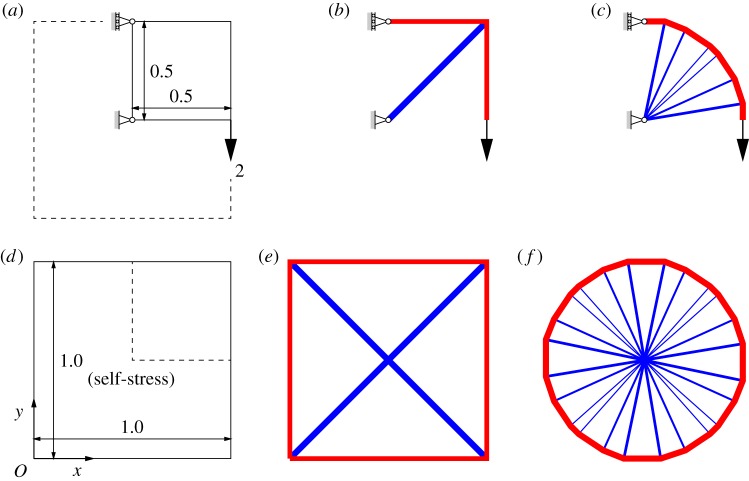
Simple truss layout optimization problems: (*a*) design domain with fixed pin and pin/roller supports and an applied load; (*b*) a solution to problem (*a*) with 2×2 nodes (*volume*=4 when *σ*^−^=*σ*^+^=1); (*c*) as (*b*) but with 13×13 nodes (*volume*=3.164, less than 1% greater than exact value of *π*); (*d*) alternative ‘self-stress’ problem; (*e*) solution to problem (*d*) with 2×2 nodes (*volume*=16) and (*f*) as (*e*) but with 25×25 nodes (*volume*=12.656, 4× the volume for problem (*c*)). (Online version in colour.)

Figure 1*a* shows the definition of a typical truss layout optimization problem, with the solutions when 2×2 nodes and 13×13 nodes are used to discretize the problem given in figure 1*b*,*c*, respectively. (In both cases, each node was inter-connected to every other node to create a ‘fully connected ground structure’, with LP then used to identify the optimum subset of truss bars). Note that, in the latter case, the solution is within 1% of the analytical optimum solution.

However, noting the observation of Denton [26] that the truss corresponding to a compatible yield-line pattern must have at least one state of self-stress (i.e. is ‘pre-stressed’), it is of interest to instead consider the closely related problem of finding the optimal layout of a truss which has no external loading (i.e. where **f**=**0**), but which is in a state of self-stress. Though this particular problem appears not to be explicitly considered in existing structural optimization literature, an appropriate mathematical formulation can tentatively be postulated. Thus, since this remains a ‘layout optimization’ problem, it seems appropriate to prescribe the state of self-stress rather loosely, for example, leaving open the possibility of many different bars being subjected to the self-stress (i.e. so as not to over-constrain the problem). This means that a single constraint can be added to give the following modified problem formulation:

2.2 $$\begin{array}{rl} \text{min}\; & V=c^{T}q\\ \text{subject to:}\; & Bq=0\\ & h^{T}q=1\\ & q\geq 0, \end{array}$$

where **h**^*T*^={*h*_1_,−*h*_1_,*h*_2_,−*h*_2_…−*h*_*m*_} and where *h*_*i*_ is a factor used to prescribe how the self-stress is to be distributed between each bar *i* (*i*=1…*m*) in the frame. Alternatively, specific bars could be allocated specific prescribed self-stress forces, if required.

A sample self-stress problem is defined in figure 1*d*, with the solutions when 2×2 nodes and 25×25 nodes given in figure 1*e*,*f*, respectively. To obtain the particular results shown, the self-stress coefficients in the constraint **h**^T^**q**=1 for each truss bar were defined by using the centre-point of the domain as a focus, achieved by using the following simple, though perhaps not intuitively obvious, rules: if the centre-point (i.e. [0.5, 0.5] in this case) lies in a vertical strip drawn directly above a given potential truss-bar *i* then coefficient *h*_*i*_ is taken as the perpendicular distance from the truss bar to the centre-point of the domain; otherwise, this is taken as zero. This gives solutions which are by inspection directly comparable to those for the problem defined in figure 1*a*, with the optimum structures shown in figure 1*b*,*c* clearly representing one-quarter of the structures shown in figure 1*e*,*f*, respectively (which are in fact simple two-dimensional *tensegrity* structures, with the former being the main part of the ‘X-shaped module’ referred to by Snelson [30], hinting at the potential for this type of problem formulation to be adapted to synthesize such structures).

It is also evident that the topology of the solution given in figure 1*f* is reminiscent of the ‘fan’-type mechanism which is critical when a slab is subjected to a point load (e.g. [5]; the numerically computed volume is also within 1% of the analytical load factor for the slab problem when a unit load is applied). In fact, it will now be demonstrated that it is this latter formulation which is directly analogous to the yield-line layout optimization problem, with the *equilibrium* truss optimization problem corresponding to the *kinematic* yield-line layout optimization problem.

### (c) Yield-line layout optimization: linear programming formulation

Maintaining precisely the same form of linear optimization problem as given in (2.2), the kinematic yield-line layout optimization formulation for an out-of-plane, quasi-statically loaded, perfectly plastic slab with supported edges and discretized using *m* nodal connections (yield-line discontinuities), *n* nodes and a single load case can be defined in equation (2.3) as follows:

2.3 $$\begin{array}{rl} \text{min}\; & E=g^{T}d\\ \text{subject to:}\; & Bd=0\\ & f_{L}^{T}d=1\\ & d\geq 0, \end{array}$$

where *E* is the energy dissipated due to rotation along the yield-lines, $d^{T}=\{\theta_{1}^{+},\theta_{1}^{-},\theta_{2}^{+},\theta_{2}^{-}\ldots \theta_{m}^{-}\}$, where $\theta_{i}^{+},\theta_{i}^{-}$ are the positive and negative relative rotations along the yield-line *i*; $g^{T}=\{m_{p1}^{+}l_{1},m_{p1}^{-}l_{1},m_{p2}^{+}l_{2},m_{p2}^{-}l_{2}\ldots m_{pm}^{-}l_{m}\}$, where *l*_*i*_, $m_{pi}^{+}$ and $m_{pi}^{-}$ are, respectively, the length and positive and negative plastic moment of resistance per unit length for potential yield-line *i*. Note that when Johansen's square yield criterion [2] is applied to isotropic slab problems, the plastic moment of resistance per unit length will be the same for all potential yield-lines, irrespective of their orientation. **B** is a suitable (2*n*×2*m*) compatibility matrix. The (relative) rotations along the yield-lines in **d** are the LP variables. (Note that for convenience the terms ‘energy dissipation’ and ‘rotation’ are here used as shorthand for ‘rate of energy dissipation’ and ‘rotation rate’, respectively.)

In this problem, **f**^T^_L_**d**=1 can be interpreted as the unit displacement constraint required in a standard virtual work formulation, where the coefficients in **f**_L_ are a function of the external live load. This ensures that the work done by the external live load is normalized, such that only the internal work done needs to be explicitly minimized in the formulation. However, it must be borne in mind that the coefficients in **f**_L_ must relate to the current problem variables, i.e. the yield-line rotations in **d**, which are *relative* rather than absolute values. Thus, the contribution to the left-hand side of the global unit displacement constraint, **f**^T^_L_**d**=1, of a given yield-line *i* will be

**Figure 2.**
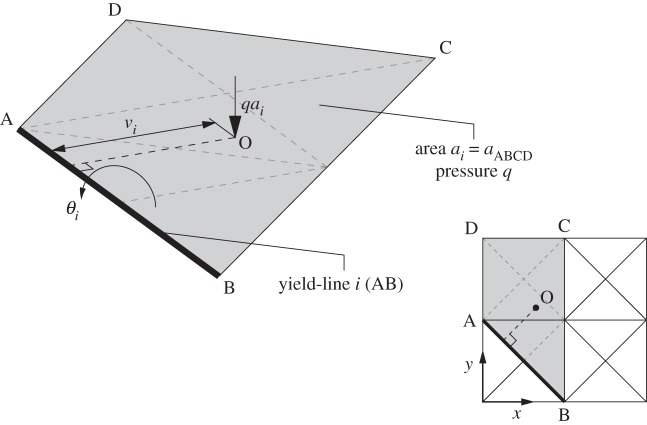
Strip ‘above’ potential yield-line *i* (AB), considered when calculating the effects of uniform live loading *q* (where O is the centroid of the strip).

2.4 $$f_{Li}^{T}d_{i}=[m_{\mathrm{Ln}i}\;-m_{\mathrm{Ln}i}]\left[\begin{array}{c} \theta_{i}^{+}\\ \theta_{i}^{-} \end{array}\right],$$

where *m*_Ln*i*_ is the moment caused by the external (unfactored) live loading on the slab. This can conveniently be calculated by considering only the effects of loads which lie in a strip of slab lying ‘above’ potential yield-line *i* (it is only necessary to work parallel to one co-ordinate axis, in this case the Cartesian *y*-axis). Thus, if it is assumed that the slab is subjected to a point load, the moment is calculated as the magnitude of the point load multiplied by the perpendicular distance to the potential yield-line. If a uniform pressure of intensity *q* is applied, then it can be seen that *m*_Ln*i*_=*qa*_*i*_*v*_*i*_, where *a*_*i*_ is the area of the strip and where *v*_*i*_ is the perpendicular distance to the centroid O of the strip, as indicated in figure 2. In summary, the use of *relative* rotations in the calculations means that the effect of a relative rotation at an individual discontinuity on the work done by the external live loads can readily be accounted for. Then, through summation over all discontinuities, the total work done by all external live loads can be determined, and then conveniently set to unity using the constraint **f**^T^_L_**d**=1.

### (d) Worked example

Consider a fixed square slab ABCD of unit area, with unit moment of resistance per unit length, and subject initially to a single central unit point load (assume vertices: A[0,0], B[1,0], C[1,1] and D[0,1]). If this problem is discretized using *n*=4 nodes, then a maximum of six potential yield-line discontinuities will interconnect the nodes, and the problem matrices and vectors of (2.3) can be written out in full as follows:

2.5 $$\begin{array}{rl} d^{T} & =[\theta_{\mathrm{AB}}^{+}\;\theta_{\mathrm{AB}}^{-}\;\theta_{\mathrm{AC}}^{+}\;\theta_{\mathrm{AC}}^{-}\;\theta_{\mathrm{AD}}^{+}\;\theta_{\mathrm{AD}}^{-}\;\theta_{\mathrm{BC}}^{+}\;\theta_{\mathrm{BC}}^{-}\;\theta_{\mathrm{DB}}^{+}\;\theta_{\mathrm{DB}}^{-}\;\theta_{\mathrm{DC}}^{+}\;\theta_{\mathrm{DC}}^{-}] \end{array}$$

2.6 $$\begin{array}{rl} g^{T} & =[1\;1\;\sqrt{2}\;\sqrt{2}\;1\;1\;1\;1\;\sqrt{2}\;\sqrt{2}\;1\;1] \end{array}$$

2.7 $$\begin{array}{rl} B & =\left[\begin{array}{cccccccccccc} 1 & -1 & \frac{1}{\sqrt{2}} & \frac{-1}{\sqrt{2}} & & & & & & & & \\ & & \frac{1}{\sqrt{2}} & \frac{-1}{\sqrt{2}} & 1 & -1 & & & & & & \\ -1 & 1 & & & & & & & \frac{-1}{\sqrt{2}} & \frac{1}{\sqrt{2}} & & \\ & & & & & & 1 & -1 & \frac{1}{\sqrt{2}} & \frac{-1}{\sqrt{2}} & & \\ & & \frac{-1}{\sqrt{2}} & \frac{1}{\sqrt{2}} & & & & & & & -1 & 1\\ & & \frac{-1}{\sqrt{2}} & \frac{1}{\sqrt{2}} & & & -1 & 1 & & & & \\ & & & & & & & & \frac{1}{\sqrt{2}} & \frac{-1}{\sqrt{2}} & 1 & -1\\ & & & & -1 & 1 & & & \frac{-1}{\sqrt{2}} & \frac{1}{\sqrt{2}} & & \end{array}\right] \end{array}$$

2.8 $$\begin{array}{rl} \text{and}\;f_{L}^{T} & =\left[\frac{1}{2}\;-\frac{1}{2}\;0\;0\;0\;0\;0\;0\;0\;0\;0\;0\right]. \end{array}$$

If the slab is instead subjected to a uniform out-of-plane pressure loading of unit intensity, the only change necessary is to replace equation (2.8) with the following equation:

2.9 $$f_{L}^{T}=\left[\frac{1}{2}\;-\frac{1}{2}\;\frac{1}{6\sqrt{2}}\;-\frac{1}{6\sqrt{2}}\;0\;0\;0\;0\;\frac{1}{6\sqrt{2}}\;-\frac{1}{6\sqrt{2}}\;0\;0\right].$$

Once the appropriate LP problems are solved, the resulting load factors at collapse can be found to be 16 and 48 for the point load and distributed load problems defined by (2.8) and (2.9), respectively. Other methods can of course be used to identify the same values for this very coarse numerical discretization, but the novel feature of the formulation described here is that *there has been no need to explicitly add a node at the centre of the slab*, something that is clearly not the case with the rigid finite-element-based methods put forward by workers such as Chan [14] and Munro & Da Fonseca [15].

In the case of the point-loaded slab, it is also evident that the solution of 16 is identical to that obtained for the ‘truss with self-stress constraints’ problem given in figure 1*e*, which is to be expected as the problems are completely equivalent mathematically. Furthermore, when more nodes are introduced the solution to the slab problem quickly approaches the exact value of 4*π* (e.g. see figure 1*f* for a solution to the mathematically equivalent truss problem). Similarly, in §5, it will be demonstrated that closer and closer approximations of the exact load factor for the uniformly loaded slab problem can be obtained as more nodes are introduced (Fox [6] identified the exact load factor for this problem to be 42.851).

### (e) Commentary

Layouts of bars in optimal ‘Michell’ trusses [31] form Hencky–Prandtl nets, which are orthogonal curvilinear co-ordinate systems (e.g. [32]). It has also been known for many years that, when Johansen's square yield criterion is employed, the layouts of yield-lines in slabs also form Hencky–Prandtl nets [33]. However, prior to the studies of the present authors, the approximate-discretized solution method developed for truss layout optimization [27] had apparently not been adapted to treat slab problems. This is despite the fact that the similarity in the form of the LP problems involved was noted many years ago by Chan [14], a talented researcher at the time active in both fields at the University of Oxford. Rectifying this situation has been the main goal of this paper.

The key features of the analogy are summarized in table 1; however, with the formulation considered thus far it is for example not yet clear how more general boundary conditions (e.g. the presence of free edges) or more complex slab geometries can be handled. The applicability of the general *discontinuity layout optimization* (DLO) formulation described by Smith & Gilbert [25,34] will therefore now be investigated.

**Table 1.** Features of analogy between truss and yield-line layout optimization problems.

	truss problem	slab problem
LP problem variables	internal bar forces in **q**	yield-line rotations in **d**
governing coefficient matrix	equilibrium: **B**	compatibility: **B**
additional constraint prescribes	self-stress	unit displacement
objective function	minimize volume *V*	minimize work *E*

## 3. Discontinuity layout optimization

### (a) General formulation

The general discretized kinematic DLO problem formulation may be stated as follows (after [25]):

3.1a $$\begin{array}{rl} \text{min}\; & \lambda f_{L}^{T}d=-f_{D}^{T}d+g^{T}p \end{array}$$

3.1b $$\begin{array}{rl} \text{subject to:}\; & Bd=0 \end{array}$$

3.1c $$\begin{array}{r} Np-d=0 \end{array}$$

3.1d $$\begin{array}{r} f_{L}^{T}d=1 \end{array}$$

3.1e $$\begin{array}{l} p\geq 0. \end{array}$$

Or alternatively as an equivalent ‘equilibrium’ formulation (derived using duality principles—e.g. [35]) as

3.2a $$\begin{array}{rl} \text{max}\; & \lambda \end{array}$$

3.2b $$\begin{array}{rl} \text{subject to:}\; & B^{T}t+\lambda f_{L}-q=-f_{D} \end{array}$$

3.2c $$\begin{array}{r} N^{T}q\leq g \end{array}$$

where λ is a dimensionless load factor, **f**_D_ and **f**_L_ are vectors, respectively, prescribing specified dead and live load effects, **d** contains displacements along the discontinuities, **B** is a suitable compatibility matrix and **N** is a suitable flow matrix. Finally, **p** and **g** are vectors of plastic multipliers and their corresponding work equation coefficients and **t** and **q** are vectors of equivalent nodal forces and forces along discontinuities, respectively.

In the kinematic formulation, the discontinuity displacements in **d** and the plastic multipliers in **p** are the LP variables, whereas in the corresponding equilibrium formulation the equivalent nodal forces in **t**, the forces along discontinuities in **q** and the load factor λ are the LP variables.

Comparing (2.3) with (3.1), the most obvious difference is that in the latter case plastic multiplier variables have been introduced, thereby effectively decoupling the compatibility and flow constraints. A consequence of this is that when duality principles are applied to obtain the dual ‘equilibrium’ formulation, the equilibrium constraint (3.2b) and yield constraint (3.2c) are properly separated.

Given that (3.1) and (3.2) only express general relations, it is now necessary to identify appropriate variables for the slab problem now being studied, starting by considering the kinematic formulation.

### (b) Kinematic formulation for slabs

Considering the kinematic problem formulation for slabs, the contributions of a given yield-line *i* to the global compatibility constraint equation (3.1b) can be written as

3.3 $$B_{i}d_{i}=\left[\begin{array}{ccc} \alpha_{i} & -\beta_{i} & 0\\ \;\beta_{i} & \alpha_{i} & 0\\ \;0 & \frac{l_{i}}{2} & 1\\ \;-\alpha_{i} & \beta_{i} & 0\\ \;-\beta_{i} & -\alpha_{i} & 0\\ \;0 & \frac{l_{i}}{2} & -1 \end{array}\right]\;\left[\begin{array}{c} \theta_{ni}\\ \theta_{ti}\\ \delta_{i} \end{array}\right],$$

where *θ*_n*i*_, *θ*_t*i*_ and *δ*_*i*_ are, respectively, the normal rotation along a potential yield-line, the twisting rotation and the out-of-plane displacement (measured at the yield-line mid-point), and where *α*_*i*_ and *β*_*i*_ are *x*-axis and *y*-axis direction cosines. Note that, unlike in (2.3), the displacement variables in **d**_*i*_ are no longer restricted to be non-negative since additional non-negative plastic multiplier variables will ensure positive energy dissipation.

Suppose that there exists no coupling between normal and twisting rotations, and between the shear displacement along a yield-line. In this case, the contributions of a given yield-line *i* to the global flow rule constraint (3.1c) can be written as

3.4 $$N_{i}p_{i}-d_{i}=\left[\begin{array}{cccccc} 1 & -1 & 0 & 0 & 0 & 0\\ 0 & 0 & 1 & -1 & 0 & 0\\ 0 & 0 & 0 & 0 & 1 & -1 \end{array}\right]\;\left[\begin{array}{c} p_{i}^{1}\\ \;p_{i}^{2}\\ \;p_{i}^{3}\\ \;p_{i}^{4}\\ \;p_{i}^{5}\\ \;p_{i}^{6} \end{array}\right]-\left[\begin{array}{c} \theta_{ni}\\ \theta_{ti}\\ \delta_{i} \end{array}\right].$$

However, at a typical yield-line, it can generally be assumed that the torsional (twisting) and out-of-plane displacements, *θ*_t*i*_ and *δ*_*i*_, respectively, will be zero, and hence these variables can be omitted from the formulation, along with their corresponding plastic multiplier variables, $p_{i}^{3},p_{i}^{4},p_{i}^{5}$ and $p_{i}^{6}$. This situation does not apply at free boundaries however, where *θ*_t*i*_ and *δ*_*i*_ should be free to take on arbitrary values, i.e. such variables should be added to signal the presence of such a boundary. This is because at a free boundary there is no limitation that the out-of-plane and torsional displacements must be zero, as would implicitly be the case if these terms were omitted from the formulation. (This makes the above formulation intrinsically more flexible than that considered in §2). Similarly, at a line of symmetry, *δ*_*i*_ should be free to take on an arbitrary value.

The objective function, (3.1a), and unit displacement constraint, (3.1d), can be formulated in a similar way to before (in §2), although now taking account of the fact that rotation normal to a yield-line is represented by a single unrestricted LP variable (the plastic multiplier variables in **p** are instead now restricted to be non-negative, ensuring the plastic dissipation **g**^T^**p** is always positive; the coefficients in **g** are as before for an internal yield-line). It should also be noted that along a free-edge (if present) **f**^T^_L*i*_={*m*_Ln*i*_,*m*_Lt*i*_,*f*_L*i*_}, and hence values for *m*_Lt*i*_ and *f*_L*i*_ will additionally need to be calculated (where *f*_L*i*_ will equal the sum of all loads lying in the slab strip ‘above’ yield-line *i* and where *m*_Lt*i*_ will equal *f*_L*i*_ multiplied by the distance between the mid-point of the yield-line and the centre of the line of action of the load in the slab strip, measured parallel to the yield-line).

### (c) Equilibrium formulation for slabs

Considering the equilibrium problem formulation for slabs, the required equilibrium constraint can be written for a potential yield-line discontinuity *i* as follows:

3.5 $$B_{i}^{T}t_{i}+\lambda f_{Li}-q_{i}=-f_{Di}$$

or, in expanded form as

**Figure 3.**
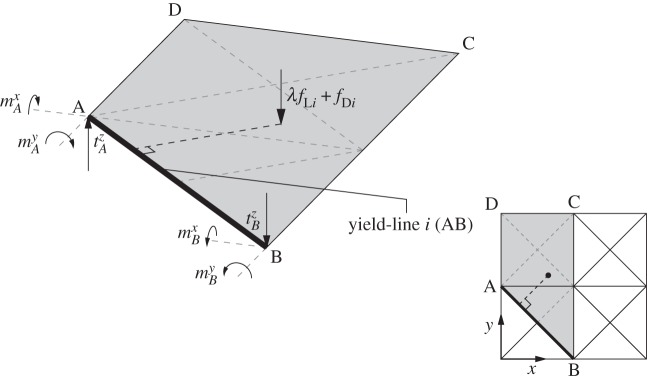
Nodal moments and forces at ends of yield-line *i* (AB), for problem shown in figure 2.

3.6 $$\left[\begin{array}{cccccc} \alpha_{i} & \beta_{i} & 0 & -\alpha_{i} & -\beta_{i} & 0\\ \;-\beta_{i} & \alpha_{i} & \frac{l_{i}}{2} & \beta_{i} & -\alpha_{i} & \frac{l_{i}}{2}\\ \;0 & 0 & 1 & 0 & 0 & -1 \end{array}\right]\;\left[\begin{array}{c} m_{A}^{x}\\ \;m_{A}^{y}\\ \;t_{A}^{z}\\ \;m_{B}^{x}\\ \;m_{B}^{y}\\ \;t_{B}^{z} \end{array}\right]+\lambda \left[\begin{array}{c} m_{\mathrm{Ln}i}\\ m_{\mathrm{Lt}i}\\ f_{Li} \end{array}\right]-\left[\begin{array}{c} M_{ni}\\ M_{ti}\\ S_{i} \end{array}\right]=-\left[\begin{array}{c} m_{\mathrm{Dn}i}\\ m_{\mathrm{Dt}i}\\ f_{Di} \end{array}\right],$$

where $m_{j}^{x}$, $m_{j}^{y}$ and $t_{j}^{z}$ can be interpreted, respectively, as *x* and *y* direction equivalent nodal moments and out-of-plane nodal force, all acting at a given node *j*, and where *M*_n*i*_,*M*_t*i*_ and *S*_*i*_ represent, respectively, the yield-line normal moment, torque and shear force acting on discontinuity *i* (figure 3). Finally, *m*_Dn*i*_,*m*_Dt*i*_,*f*_D*i*_ and *m*_Ln*i*_,*m*_Lt*i*_,*f*_L*i*_ represent the dead and live load effects acting at discontinuity *i*.

Now considering the contribution of a given yield-line *i* to the global yield constraint (3.2c), initially assuming that *N*_*i*_ is as defined in equation (3.4)

3.7 $$N_{i}^{T}q_{i}=\left[\begin{array}{ccc} 1 & 0 & 0\\ -1 & 0 & 0\\ 0 & 1 & 0\\ 0 & -1 & 0\\ 0 & 0 & 1\\ 0 & 0 & -1 \end{array}\right]\;\left[\begin{array}{c} M_{ni}\\ M_{ti}\\ S_{i} \end{array}\right]\leq \left[\begin{array}{c} m_{pi}^{+}\\ \;m_{pi}^{-}\\ \;m_{ti}^{+}\\ \;m_{ti}^{-}\\ \;s_{i}^{+}\\ \;s_{i}^{-} \end{array}\right].$$

Although at a typical yield-line, inequality equation (3.7) reduces simply to $m_{pi}^{-}\leq M_{ni}\leq m_{pi}^{+}$, by inspection it is clear that more complex yield functions could be introduced if required, for example involving interaction between the normal and torsional moments (though in doing so the traditional ‘yield-line’ character of the solution is likely to be lost, e.g. a twisting failure would lead to loss of contact between the two ends of the parts of a slab adjoining a given yield-line).

## 4. Extensions to the basic discontinuity layout optimization procedure

### (a) Treating non-convex problem domains

Although the benchmark plane strain metal-forming and geotechnical problems considered in Smith & Gilbert [25] all had simple rectangular problem domains, real-world slab-geometries will often be considerably more complex, e.g. comprising complex non-convex problem domains. Although such geometries present no particular difficulties for conventional finite-element-based formulations, various issues arise when the DLO procedure is applied. These will now be explored.

#### (i) Inter-nodal connections in non-convex problem domains

**Figure 4.**
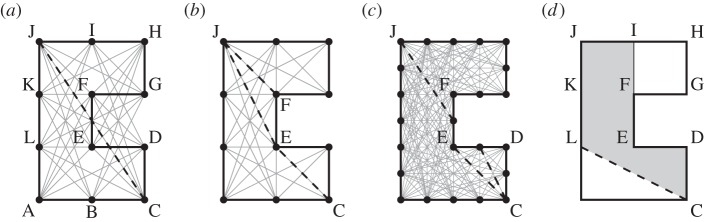
Slab with non-convex ‘C’-shaped geometry: (*a*) potential yield-lines, with critical yield-line CJ which crosses domain boundaries highlighted; (*b*) reduced set of potential yield-lines with alternatives to CJ highlighted; (*c*) as (*b*) but with finer nodal spacing along edges and (*d*) shaded area to be considered when formulating **f**_L_ and **f**_D_ terms for potential yield-line CL.

Consider the non-convex slab (ABCDEFGHIJKL) shown in figure 4. If it is assumed that each node is connected to every other node by potential yield-lines, then it is evident that some potential yield-lines (e.g. the highlighted yield-line CJ in figure 4*a*) cross ‘free space’. To address this, it has been found to be convenient to disallow such potential yield-lines. However, since this means that a good representation of a previously well represented possible mode of response may then not be achievable (e.g. figure 4*b*), a finer nodal discretization can be used along all boundaries to partially compensate for this, figure 4*c*; consequently in all example problems considered herein the nodal spacing along boundaries has been set to be half that used within the interior of a slab.

#### (ii) Computing load effects in non-convex problem domains

It is also necessary to consider how the load terms in **f**_L_ and **f**_D_ should be computed when a non-convex slab is involved. Thus, referring to figure 4, suppose that the slab has material properties, support and loading conditions which mean that, at failure, part of the slab (CDEFGHIJKL) rotates as a rigid element about a single yield-line CL, i.e. as indicated in figure 4*d*. Assuming both dead and live loads are involved, it is instructive to consider how the components in **f**_L*i*_ and **f**_D*i*_ can be calculated for *i*=*CL*. In this case, as only the area shaded (CDEFIJKL) will be directly influenced by rotation along CL, only loading within this shaded area need be accounted for in the calculations. The remaining unshaded area lying ‘above’ potential yield-line CL (i.e. area FGHI) will clearly also move in the mechanism postulated, but the work associated with this movement will be accounted for through displacement along edge FG (combined translation and rotation), with the relative displacements at the edge of the slab in effect being absolute displacements.

### (b) Simplifying complex yield-line patterns

It was pointed out earlier in the paper that the layouts of yield-lines in slabs will, like bars in optimal trusses, take the form of Hencky–Prandtl nets, which are orthogonal curvilinear co-ordinate systems. A side-effect of this is that it will frequently be found that the true critical failure mechanism will include one or more areas comprising an infinite number of infinitely short yield-lines. Although strictly speaking correct, such mechanisms do not appear to be in the spirit of the original yield-line analysis method, and the presence of large numbers of yield-lines can also make visualization of the collapse mechanism and hand checking of solutions difficult; the latter is potentially very important in engineering practice. (Furthermore, considering application to reinforced concrete slabs, cracks tend in reality to be discrete and spaced of the order of centimetres apart in yielding regions, owing to the finite tensile strength of the concrete.)

A practical means of simplifying the yield-line patterns identified is to use a coarse nodal refinement (e.g. compare the simple layout of figure 1*b* with that of figure 1*c*). However, this means that there is a danger that important detail will be lost. Thus, the efficacy of a method which involves penalizing short yield-lines in order to simplify failure mechanisms will be investigated. Such a method appears to have been first proposed by Parkes [36], though in the context of truss layout optimization.

In essence, this method only requires that $g^{T}=\{m_{p1}^{+}l_{1},m_{p1}^{-}l_{1},\ldots m_{pm}^{-}l_{m}\}$ is replaced with ${\hat{g}}^{T}=\{m_{p1}^{+}(l_{1}+k),m_{p1}^{-}(l_{1}+k),\ldots m_{pm}^{-}(l_{m}+k)\}$ when formulating the optimization problem, where *k* is a value designed to give the desired level of simplification. Then, once the optimization process is complete, a corrected computed load factor can be obtained by back-substituting the original values from **g** into the objective function equation (assuming the kinematic formulation is being used). The efficacy of this approach will be explored for the example problems considered in §5.

## 5. Examples

The procedure will now be applied to a range of isotropic slab problems previously studied in the literature, including some which have known analytical solutions.

### (a) Computational issues

To obtain the solutions, a workstation equipped with an Intel Xeon E5-2670 CPU and running 64-bit CENTOS Linux was employed. The Mosek commercially available interior point LP optimizer, which uses the homogeneous and self-dual algorithm, was used [37]. The problem was initially passed to the optimizer in memory and subsequently only changes to the current problem needed to be passed to the optimizer, rather than the entire revised problem. The pre-solve feature of the optimizer was enabled and default tolerances were used. In all cases, nodes were distributed on a uniform Cartesian grid with the specified number of nodal divisions being the number used across a specified length of the interior of a given slab. The number of nodal divisions used along exterior edges was twice that used within the slab interior, as described in §4*a*.

#### (i) Adaptive nodal connection scheme

To significantly increase the size of problem which could be solved, the adaptive nodal connection procedure, described by Gilbert & Tyas [29] for layout optimization of trusses, and in the context of DLO by Smith & Gilbert [25], was used when solving all problems. Using this procedure, it is only necessary to connect adjacent nodes with potential discontinuities initially, with additional potential discontinuities then added as required (a simple check for yield violation is carried out following an LP iteration to decide whether further potential discontinuity connections need to be added, and hence whether a further LP iteration is required). In the examples considered here, it was specified that not more than 5% of the number of connections present in the initial, adjacent connectivity, problem could be added at each iteration. Even though changes to the LP problem at each iteration might be relatively modest, with the interior point optimizer used it was not possible to use the solution from a previous iteration as a starting point for the next optimization (i.e. a ‘warm start’ was not used). Additionally, although the adaptive procedure is amenable to parallelization, and a parallel version of the Mosek optimizer is available, a single processor was used for all computations. The CPU times quoted include only the time to solve the LP problem(s); in practice, some additional time is required to identify candidate connections for admission at the next iteration in the adaptive solution procedure used.

#### (ii) Treating overlapping discontinuities

The greatest common divisor algorithm referred to in Smith & Gilbert [25] was used to remove overlapping potential discontinuities, except when the simplification algorithm outlined in §4*b* was used (since this requires overlapping potential discontinuities to be present in order to work effectively).

### (b) Square slabs with known exact solutions

**Figure 5.**
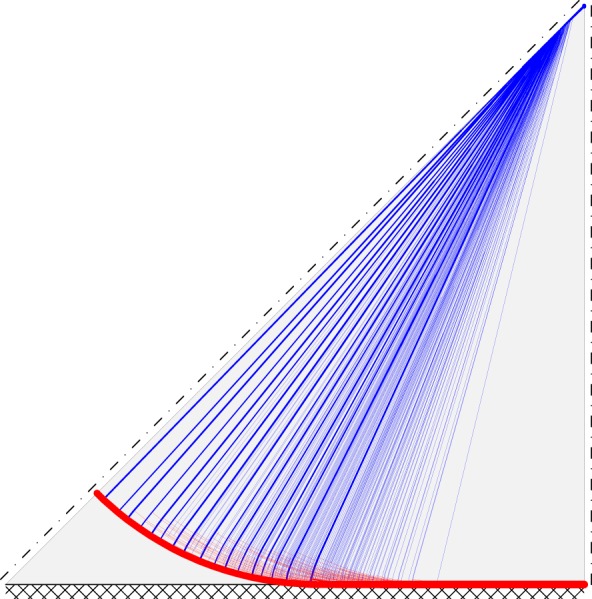
Square slab with fixed supports: DLO yield-line pattern (320 nodal divisions). (Online version in colour.)

Initially consider a square slab of side length *L* which is subjected to uniform pressure loading *q* and which has a plastic moment of resistance per unit length of *m*_p_. If the support type around the perimeter is unchanging, then symmetry conditions mean that only one-eighth of the slab needs to be modelled. DLO solutions and corresponding CPU times for slabs with fixed and simple supports are shown in table 2, for various nodal discretizations. Figure 5 shows the solution for the fixed support case when using the finest nodal discretization, involving 320 nodal divisions.

**Table 2.** Square slabs with known exact solutions: numerical versus analytical solutions.

support type	analytical λ(*m*_*p*_/*qL*^2^)	nodal divisions^*a*^	numerical λ(*m*_p_/*qL*^2^)	error%	CPU (s)
simple	24.0	1	24.000	0.000	<1
fixed	42.851	1	48.000	12.016	<1
	[6]	20	43.055	0.476	2
		40	42.934	0.194	66
		60	42.908	0.133	278
		80	42.887	0.085	1105
		100	42.879	0.064	1704
		120	42.874	0.054	4835
		140	42.870	0.045	15 655
		160	42.868	0.040	54 949
		180	42.865	0.033	71 420
		200	42.863	0.028	276 301
		220	42.862	0.025	594 702
		240	42.861	0.023	855 442
		260	42.860	0.021	1 299 532
		280	42.859	0.018	985 247
		300	42.858	0.016	1 695 220
		320	42.857	0.015	912 559
		$\infty^{b}$	42.851	0.000	—

^*a*^Number of divisions along each leg of the right-angled triangle domain analysed.

^*b*^ Extrapolated value (see appendix A for extrapolation procedure).

When simple supports are present the exact solution (λ=24.0(*m*_p_/*qL*^2^)) can be obtained when only three nodes are present (i.e. at the corners of the portion of slab being modelled). Increasing the total number of nodes therefore does not change the solution in this case.

For the fixed support problem, it is evident from table 2 that the DLO procedure can obtain a solution which is within 0.5% of the exact analytical solution in only 2 s. This is in contrast to previously proposed automated yield-line analysis methods, which have struggled to obtain accurate solutions for this particular problem without recourse to specially tailored meshes. The best solution obtained for the fixed support problem (42.857(*m*_p_/*qL*^2^) is just 0.015% higher than the exact solution (42.851(*m*_p_/*qL*^2^)), though in this case the CPU time required was long (912 559 s). The solutions obtained using nodal divisions of between 20 and 320 were used to calculate an extrapolated solution (refer to appendix A for details of the extrapolation method used). The extrapolated solution was found to be 42.851(*m*_p_/*qL*^2^), which matches the exact solution quoted by Fox [6] to all five significant figures, indicating that the DLO procedure can, if required, be used to obtain extremely accurate numerical solutions.

**Figure 6.**
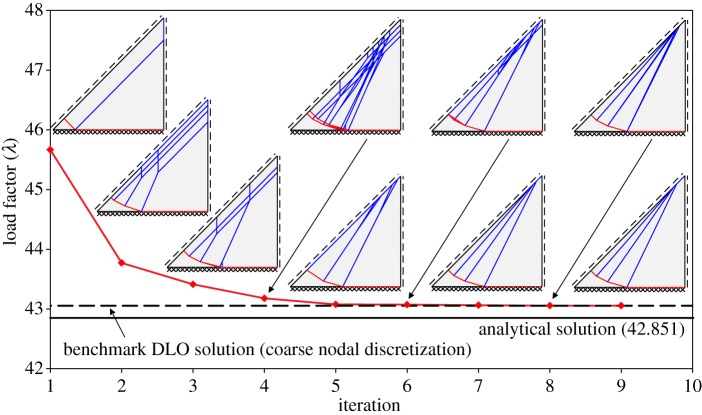
Square slab with fixed supports: numerical solution versus iteration when using adaptive nodal connection scheme (20 nodal divisions). (Online version in colour.)

Finally, figure 6 shows how the computed collapse load and associated mechanism changes as the adaptive nodal connection procedure employed proceeds, here using a coarse nodal discretization involving 20 nodal divisions for sake of clarity.

### (c) Regan and Yu's irregular slabs

**Figure 7.**
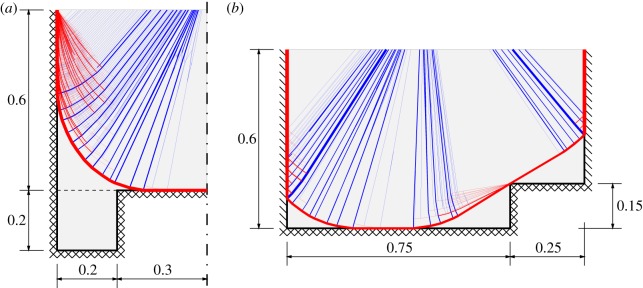
Regan and Yu's irregular slabs: (*a*) with alcoves and (*b*) indented, showing geometries and DLO solutions (120 nodal divisions). Simple and fixed supports are denoted, respectively, by single and cross hatches. (Online version in colour.)

The next two slab problems were originally included in the book by Regan & Yu [38] and are somewhat more complex, with varying support conditions and non-convex geometries. Both the ‘slab with alcoves’ and ‘indented slab’ problems involve a pressure load of unit intensity and unit plastic moment of resistance per unit length. The geometries of the slabs and sample DLO solutions are presented in figure 7.

In table 3, solutions obtained by previous workers are presented alongside new DLO results. It is clear that even the coarsest DLO solutions presented (involving 20 nodal divisions) improve upon (i.e. are lower than) previously obtained upper-bound solutions. This is despite the fact that some of the previously obtained numerical solutions benefitted from the use of problem-specific element meshes, tailored to yield the best possible solutions. The DLO solutions are also bracketed by the upper and lower bound solutions computed by Jackson [21].

**Table 3.** Regan and Yu's irregular slabs: literature solutions versus DLO solutions.

reference	bound	nodal divisions^*a*^	slab with alcoves λ	indented slab λ
Regan & Yu [38]	upper	—	41.6^*c*^	33.3^*c*^
Johnson [39]	upper	—	37.0	32.5
Thavalingham *et al*. [18]	upper	—	35.8	29.2
Jackson [21]	upper	—	35.8	29.2
	lower	—	35.1	28.5
DLO	upper	20	35.589	29.174
	upper	40	35.411	29.062
	upper	60	35.330	29.034
	upper	80	35.305	29.014
	upper	100	35.293	29.010
	upper	120	35.279	29.002
	upper	140	35.267	28.998
	upper	160	35.262	28.995
	upper	180	35.257	28.995
	upper	200	35.254	28.992
	upper	220	35.251	28.991
	upper	240	35.250	28.990
	upper	260	35.247	28.990
	upper	280	35.245	28.989
	upper	300	35.244	28.988
	upper	320	35.243	28.988
	upper	340	35.243	—
	upper	360	35.242	—
	upper	380	35.241	—
	—	$\infty^{b}$	35.230	28.980

^*a*^Number of divisions per unit length (i.e. the total length of each of the slabs, neglecting symmetry).

^*b*^Extrapolated values, obtained using the 16 most refined solutions (see appendix A for extrapolation procedure).

^*c*^Computed using the yield-line patterns shown in Regan & Yu [38]; these values are slightly lower than the simplified finite-element mesh derived solutions quoted by Johnson [39].

### (d) Slab with hole

**Figure 8.**
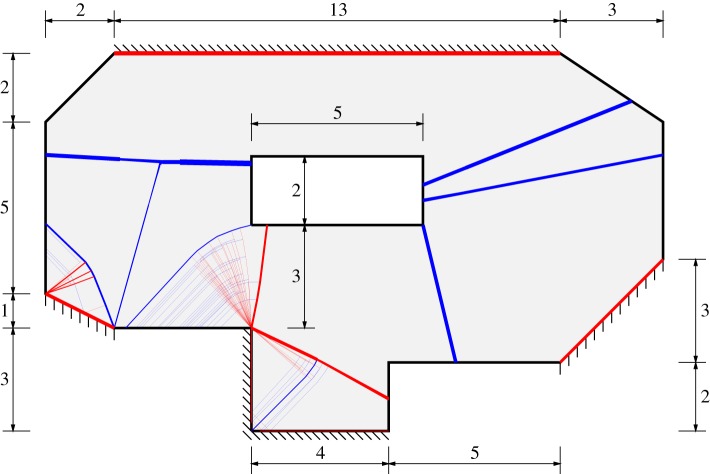
Slab with hole: geometry (dimensions in metre) and DLO solution (120 nodal divisions). (Online version in colour.)

The final example considered comprises the irregular polygonal slab containing a hole previously analysed by Olsen [40], Krabbenhøft *et al.* [10] and others. Here, the slab is assumed to be isotropic with unit plastic moment of resistance per unit length and is subjected to a pressure load of unit intensity. The slab geometry and DLO solution are shown in figure 8. The computed DLO load factor was found to be 0.13554, which is bracketed by the upper and lower bound solutions reported by Jackson [21], as indicated in table 4. Also, the solution is 0.4% higher than the approximate lower bound solution reported by Krabbenhøft *et al.* [10]. This example demonstrates that the DLO procedure can be applied to problems with realistic geometries, something that is essential for industrial application.

**Table 4.** Slab with hole: literature versus DLO solutions.

reference	bound	nodal divisions^*a*^	solution λ
Jackson [21]	upper	—	0.137
	lower	—	0.132
Krabbenhøft *et al*. [10]	lower (approx.)	—	0.135^*b*^
DLO	upper	120	0.13554

^*a*^Number of divisions per 10 m slab length.

^*b*^Calculated by dividing the quoted pressure load (6.75) by the quoted plastic moment of resistance (50).

### (e) Simplified solutions

**Figure 9.**
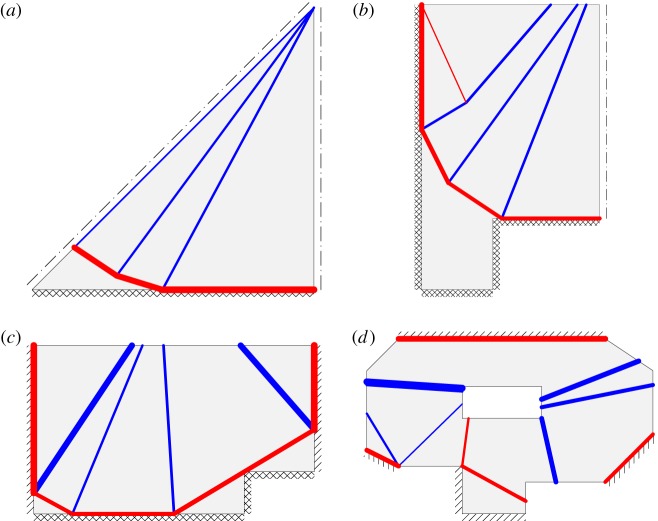
Simplified failure mechanisms: (*a*) fixed square slab (40 nodal divisions, *k*=0.005, λ=43.080 (diff: 0.53%)); Regan & Yu's (*b*) slab with alcoves (40 nodal divisions, *k*=0.02, λ=35.852 (diff: 1.77%)) and (*c*) indented slab (40 nodal divisions, *k*=0.05, λ=29.293 (diff: 1.08%)); (*d*) slab with hole (50 nodal divisions, *k*=0.5, λ=0.13640 (diff: 0.63%)). (Differences relative to (*a*) analytical solution given in table 2, (*b*), (*c*) extrapolated DLO solutions given in table 3, and (*d*) numerical DLO solution given in table 4.) (Online version in colour.)

**Figure 10.**
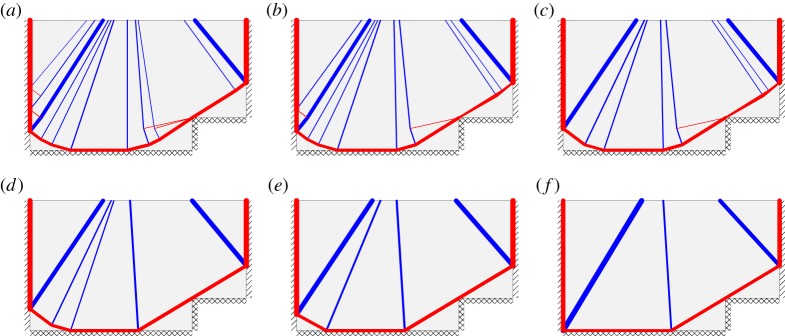
Regan and Yu's indented slab: influence of simplification factor *k* on DLO solution, using 40 nodal divisions. (*a*) *k*=0, λ=29.062 (diff: 0.28%), (*b*) *k*=0.001, λ=29.067 (diff: 0.30%), (*c*) *k*=0.002, λ=29.104 (diff: 0.43%), (*d*) *k*=0.005, λ=29.205 (diff: 0.78%), (*e*) *k*=0.05, λ=29.293 (diff: 1.08%) and (*f*) *k*=0.1, λ=29.965 (diff: 3.40%). (Differences relative to extrapolated DLO solution given in table 3.) (Online version in colour.)

It is evident from the preceding examples that many of the DLO solutions identified are rather complex, and distinctly different to the ‘textbook’ yield-line solutions most practicing engineers are familiar with (for reasons which will be briefly discussed in the next section). However, by using the procedure described in §4*b*, simpler, more familiar looking, yield-line patterns can be generated. Sample simplified solutions for each of the examples considered are shown in figure 9; values for the simplification factor *k* were chosen on a case-by-case basis to provide the desired level of simplification. Figure 10 shows how the value of *k* influences the yield-line pattern for Regan and Yu's indented slab example.

It is evident that simplified yield-line patterns can successfully be generated, and, although the corresponding load factors are somewhat less accurate than calculated using the standard DLO procedure, they are mostly very similar, demonstrating that the load factor is often relatively insensitive to the precise form of the collapse mechanism. Also, the efficacy of the simplification technique is likely to depend on the type of problem being considered.

## 6. Discussion

Developing a procedure to automatically identify upper bound limit analysis solutions has been of interest to researchers for many decades. In the case of slabs, a number of researchers have proposed procedures designed to improve upon the solution obtained using an initial rigid finite-element analysis (e.g. obtained using the method described by Munro & Da Fonseca [15]), by refining this in a subsequent iterative nonlinear optimization phase (e.g. [17,18]). In fact, when the adaptive nodal connection scheme described in §5*a* is employed, the initial solution obtained using the DLO procedure will be precisely the same as that obtained using rigid finite elements (assuming nodes are identically positioned in both cases, and assuming nearest neighbour connectivity in the case of DLO). What is new here is that when DLO is used the form of the yield-line pattern is permitted to change completely at subsequent iterations (e.g. to a fan mechanism). Additionally, the convex nature of the underlying mathematical optimization problem is preserved, and, even when the adaptive nodal connection procedure is used, the solution obtained will be globally optimal for the prescribed nodal discretization. This demonstrates that the widely held belief that recourse to nonlinear, non-convex, mathematical optimization procedures is necessary in order to directly identify critical yield-line patterns is misplaced. The DLO procedure also appears to retain much of the elegant simplicity of the original yield-line analysis method. Compared with more conventional finite-element limit analysis methods (e.g. [10]), the underlying formulation is simpler and involves only linear constraints when Johansen's square yield criterion is involved. Furthermore, visual interpretation of the output is straightforward as discrete yield-lines can clearly be identified.

**Figure 11.**
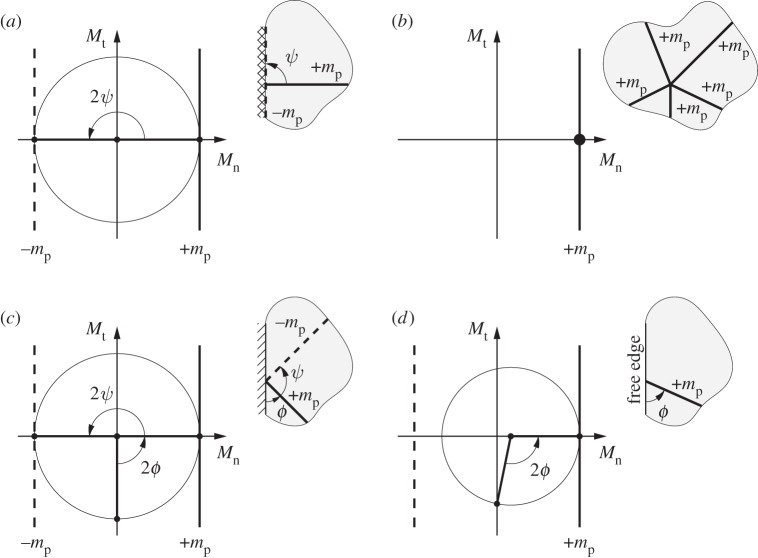
Use of Mohr's circles in normal moment (*M*_n_)–torque (*M*_t_) space to illustrate characteristic features of critical yield-line patterns in isotropic slabs: (*a*) orthogonal intersection of yield-lines of opposite sign, here at a fixed edge; (*b*) intersection of yield-lines of the same sign at arbitrary angles; (*c*) intersection of yield-lines of opposite sign at simple support (where *ϕ*=45° if $m_{p}=m_{p}^{+}=m_{p}^{-}$) and (*d*) yield line intersecting a free edge (at 45° ≤*ϕ*≤135° if $m_{p}=m_{p}^{+}=m_{p}^{-}$).

High-resolution DLO solutions also allow a number of characteristic features of critical yield-line patterns in isotropic slabs to be observed, which can readily be confirmed via the use of Mohr's Circles. For example:

— yield-lines of opposite signs should intersect at 90°, whether in the interior of a slab or at a fixed support, as indicated in figure 11*a*;
— yield-lines of the same sign can intersect at any angle, as indicated in figure 11*b*;
— yield-lines of opposite signs should intersect simple supports at 45° and 135° (when $m_{p}=m_{p}^{+}=m_{p}^{-}$), as indicated in figure 11*c*; and
— yield-lines should intersect free edges at between 45° and 135° (when $m_{p}=m_{p}^{+}=m_{p}^{-}$), figure 11*d*. (Note that, as pointed out by Nielsen & Hoang [41], Kirchhoff boundary conditions permit a torsional moment to exist along a free edge. Thus, it is not necessary for critical yield-lines to intersect free edges at 90°, as suggested by Quintas [42]).

These characteristic features are generally not enforced when postulating simple yield-line patterns, either by hand or when using low numbers of nodes with DLO, and strictly would only be fully enforced when using an infinite number of infinitesimally spaced nodes. Since solutions generated using high numbers of nodes will often lead to highly complex patterns, a simplification procedure has also been presented, which provides a pragmatic means of identifying less complex layouts. A potential practical advantage of such layouts is that they can be used to generate traditional engineering calculations, which can readily be checked by hand by a practitioner.

## 7. Conclusion

(i) In this paper, it has been demonstrated that the problem of identifying critical yield-line patterns can be formulated as a simple, albeit relatively large-scale, LP problem. This overturns the widely held belief that recourse to complex nonlinear, non-convex, mathematical optimization procedures is necessary in order to directly identify critical yield-line patterns.
(ii) The analogy between approximate-discretized formulations for truss layout optimization and yield-line layout optimization has been established. The DLO procedure used retains much of the inherent simplicity of the traditional hand-based yield-line analysis method. Excellent agreement with known exact solutions has been obtained and improved solutions to a number of problems described in the literature have been obtained.
(iii) Unlike previously proposed upper bound computational limit analysis methods, the DLO procedure presented can identify ‘fan-type’ yield-line mechanisms, as well as mechanisms of any other geometry. The procedure therefore appears to be the first *truly systematic* analysis tool capable of directly identifying yield-line patterns to have been developed to date.
(iv) The yield-line patterns identified by the DLO procedure are often observed to be complex, containing numerous closely spaced yield-lines. However, it is shown that such complex failure mechanisms can be simplified if required (e.g. to facilitate hand-checking), albeit at the expense of some accuracy.

## Figures and Tables

**Figure 12.**
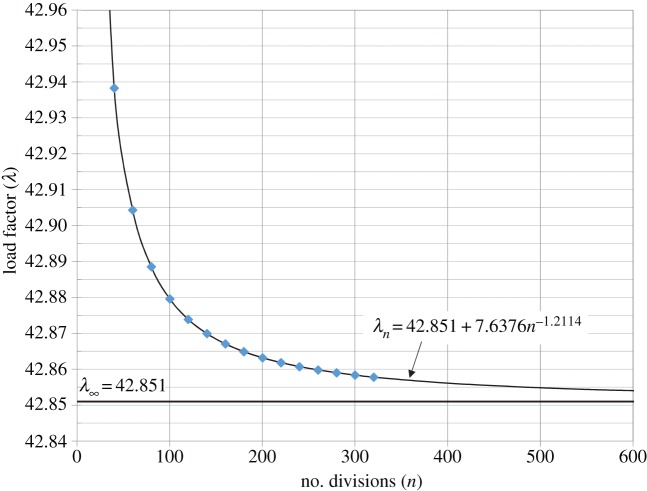
Square slab with fixed supports: computed load factor versus nodal refinement. (Online version in colour.)

## Appendix A. Computing extrapolated load factors

In common with truss layout optimization problems (e.g. [43]), the solutions obtained using the proposed layout optimization procedure appear to follow a relation of the form:

A 1 $$\lambda_{n}=\lambda_{\infty }+kn^{-\alpha },$$

where λ_*n*_ is the numerically computed load factor for *n* equally spaced nodal divisions, $\lambda_{\infty }$ is the load factor when n→∞, and *k* and *α* are positive constants. Using (A 1), a weighted nonlinear least-squares approach can be used to find best-fit values for $\lambda_{\infty }$, *k* and *α*, with the weighting coefficient taken as *n*. For example, the resulting trend line and value for $\lambda_{\infty }$ for the fixed edge square slab are shown in figure 12.
